# Square dance, loneliness, quality of life, and attitude toward aging in middle-aged and older women in China

**DOI:** 10.3389/fpubh.2025.1508556

**Published:** 2025-02-21

**Authors:** Ruitong Li, Qin Yan, Yujia Qu, Yan Wang

**Affiliations:** College of Education, Beijing Sport University, Beijing, China

**Keywords:** square dance, middle-aged and older women, quality of life, loneliness, attitude toward aging

## Abstract

**Background:**

With the ongoing advancement of society and the economy, population aging has emerged as an unavoidable global trend, leading to various social issues and exerting a profound impact on the physical and mental well-being of middle-aged and older adults. While women often face greater challenges than men during the aging process, particularly in terms of physical health vulnerabilities, mental health disparities and socioeconomic disadvantages. These factors underscore the importance of studying middle-aged and older women to better address their unique needs and promote healthy aging. Much attention has been paid to how middle-aged and older women can improve healthy aging, positive emotions and subjective well-being through regular physical activity.

**Methods:**

Using 4,819 middle-aged and older women who participated in square dance exercise, structural equation modeling was used to explore the relationship between square dance exercise and attitude toward aging, as well as the chain-mediated effects of loneliness and quality of life.

**Results:**

(a) Square dance exercise is a positive predictor of attitude toward aging in middle-aged and older women. (b) Loneliness and quality of life moderated the relationship between square dance exercise and attitude toward aging, and their mediating effects included three pathways. (c) The single mediating effect of quality of life was the largest compared to the chain mediating effects of loneliness and loneliness-quality of life.

**Conclusion:**

This study aimed to reveal the reduction of loneliness and improvement of quality of life as potential mechanisms in the relationship between square dance exercise and attitude toward aging, and to emphasize that square dance exercise can be effective in promoting positive attitude toward aging and enhancing subjective well-being in middle-aged and older women.

## 1 Introduction

As population aging leads to an increase in the number of older persons in all societies, the promotion of healthy or successful aging has become an issue of considerable importance (1). China stands out, however, as it already has the world’s largest older population (2). According to the World Health Organization (WHO), healthy aging is more than just the absence of disease, it is the process of developing and maintaining the functional ability that enables well-being in older individuals (3). A lack of physical activity in middle-aged and older adults may lead to deterioration in mental health as they age, as well as a decline in the body’s overall functional capacity (4). At the same time, the lack of perceived physical abilities and the complex health problems associated with gradual aging can also trigger a sense of loneliness in middle-aged and older adults, thus interfering with their quality of life (5). Participation in leisure-time physical activity can serve as a means for middle-aged and older adults to effectively cope with aging, reduce negative psychological symptoms, including depression and anxiety, and improve their mental health and quality of life (6).

Physical activity is globally recognized as a vital health asset for all ages and serves as a non-pharmacological intervention to prevent and treat numerous diseases. In China, square dancing, also known as square fitness dance, has become an increasingly popular form of exercise. It involves unified group dances performed spontaneously to music in public spaces such as squares, parks, streets and open areas near buildings (7). Compared to traditional sports like running or swimming, square dancing is simple, beginner-friendly and flexible, as it is not restricted by time, theme or rhythm. This group-based, music-guided outdoor activity has been found to better promote mental health than indoor or individual sports like yoga or tennis (8).

Square dancing is widely practiced across the country, with participants primarily consisting of middle-aged and older women aged 40–65. Role theory indicates that women in this age group often experience emotional and physical challenges stemming from role transitions. As they adapt to changing social roles, square dancing provides them with a platform to assert their individuality, express themselves and remain active participants in urbanized society. As such, it has become a meaningful way for women to reconstruct social relationships and rebuild their sense of identity (9).

Emerging alongside China’s rapid urbanization following its reform and opening-up, square dancing has evolved into a significant cultural phenomenon. Ecosystem theory (EST) suggests (10) that individuals are shaped by various interconnected social systems directly or indirectly, and has been applied to studies in recent years on successful aging. Many researchers attribute the rise and popularity of square dancing to broader social changes. As a cultural activity, square dancing incorporates elements of urban, rural, community, fitness, leisure and national cultures, enriching the fabric of contemporary Chinese social culture.

In summary, the focus on middle-aged and older women arises how they often face challenges such as role transitions, changes in health status and weakened social support systems. Square dancing not only helps them address these issues but also fosters social connections, enhances life satisfaction and improves mental health.

According to the World Social Report 2023, women are becoming the mainstay of middle-aged and older adults, especially older adults (11). Square dance exercise has become the first choice for Chinese middle-aged and older women to participate in physical activity (12). Regular square dance exercise can improve numerous psychological indicators in middle-aged and older women, effectively reduce loneliness, increase life satisfaction, and generate an optimistic and positive attitude toward aging (13). Therefore, based on previous studies, square dancing can be an important means to improve the quality of life, reduce loneliness and promote active aging among middle-aged and older adults. However, as a unique form of exercise for middle-aged and older adults in China, the relationship between square dancing and aging attitudes in this population has not been fully confirmed. However, the direct effects of square dancing on the aging attitudes of middle-aged and older adults, as well as the role of loneliness and quality of life as mediating variables, have not been fully investigated in the middle-aged and older population.

Therefore, in order to fill this research gap, this study selected loneliness and quality of life as mediator variables from the perspective of psychology to explore the relationship between square dance and the aging attitudes of middle-aged and older adults. It is hoped that through a large-sample cross-sectional study, the chain-mediated effects of loneliness and quality of life will be verified, the relationship between square dancing and aging attitudes of middle-aged and older people and its internal mechanism will be elucidated, so as to provide research bases and theoretical references for the promotion of healthy aging.

### 1.1 Square dance and attitude toward aging

Attitude toward aging are expectations about one’s experience and evaluations of the process of aging and of growing old (14). Attitude toward aging are divided into positive and negative attitudes based on middle-aged and older adults’ feelings. Positive attitude toward aging are positive experiences and feelings that are related to health and well-being. Negative attitude toward aging stem from declines in physical, mental, and social capabilities (15). When middle-aged and older women engage in physical activities, they make relatively positive evaluations of their own life events, and their sense of efficacy and positive thoughts become the basis of their attitudes toward life (16).

Attitudes toward physical functioning and emotional well-being can have long-term effects on one’s own aging (17). Social–emotional choice theory suggests that as middle-aged and older women grow older, the accumulation of knowledge and experience encourages them to better regulate their emotions and to find things in their living environment that are conducive to regulating their emotions to release them (18). Physical activity can help middle-aged and older adults achieve good physical function and positive emotional experiences, prevent disease, promote mental health, and effectively improve attitude toward aging (19). When middle-aged and older women perceive stressors in their bodies, they can focus on upbeat music to release the tension and pressure, effectively transferring and dispersing their negative emotions (20). First, in a study comparing dance, listening to music, and cycling in non-clinical populations, both dancing and passive listening to music were shown to enhance positive emotions, reduce negative emotions and fatigue. Meanwhile, regular voluntary participation in exercise can reduce emotional inertia (21). Second, compared with the square dance practitioners, Tai Chi practitioners have better physical health and immune function (22). Third, square dancing has a greater positive impact on mental health and illness than walking exercise, table tennis and running (23). As can be seen from the above that square dance exercise effectively reduced the dimension of psychosocial loss, improved the dimension of physical change, enhanced the sense of psychological gain and further improved the mental health of middle-aged and older individuals. Lastly, square dance exercise has a positive impact on all aspects of their physical fitness. Square dance exercise can effectively regulate the body composition of middle-aged and older women, improve their physical fitness and prevent physical function problems that occur as they grow older (24). The muscle strength of the lower limbs increases during exercise, the dynamic balance of the body improves, osteoporosis is prevented, and the health of the shoulder joints is protected (25).

In summary, physical activity can foster a positive outlook on aging, which in turn significantly impacts their mental health (26). Square dance exercise, in particular, helps improve attitude toward aging and contributes to participants’ psychological well-being.

### 1.2 Loneliness and attitude toward aging

Loneliness is the gap between one’s expectations of social relationships and actual social relationships, including both emotional loneliness due to loss or lack of close emotional attachments and social loneliness due to infrequent contact or lack of engagement (27). Loneliness is considered an adaptation that can facilitate interpersonal and social relationships (28). Among those aged ≥55 years, 22% of the female population experiences loneliness, and this proportion rises with increasing age (29). At the same time, aging-related physical incapacity and complex health problems are more likely to trigger loneliness in middle-aged and older women (5). Loneliness is a key factor affecting active aging. Physical activity can provide enriching opportunities for middle-aged and older women experiencing loneliness to develop positive and meaningful social relationships with other participants, leading to positive emotions in old age (30). However, poor health or the need to care for an ill spouse or other relative can limit physical activity and social interaction, thereby increasing the risk of loneliness. Instead, it can have a negative impact on the survival and overall well-being of older persons (31).

Square dance exercise is a group physical activity that is essential for experiencing a sense of belonging and social identity, and helps to improve the well-being of middle-aged and older women (32). Meanwhile, social interaction theory suggests that social activities are important for improving the psychological and social adaptability as well as enhancing the physical and mental health of middle-aged and older adults (33). High-quality participation in social activities expands the range of social activities of older persons, increases social interactions, maintains and strengthens intimate relationships, thereby reducing loneliness and improving the quality of life in old age (34). Square dancing, as a recreational physical activity, is believed to create a rich social interaction environment for participants, and the better the social interaction environment, the more it helps middle-aged and older women achieve successful aging (35). Square dancing also promotes their social interactions with peers, maintains social engagement, eliminates loneliness, and ultimately promotes psychosocial health experiences (36).

In conclusion, the more middle-aged and older women interact with their peers and groups through square dance exercise, the more connected they become to others and society. Therefore, loneliness is improved.

### 1.3 Quality of life and attitude toward aging

The WHO recommends that the goal of achieving an “active aging” policy framework should be to improve the quality of life of older persons (37). Quality of life is a multidimensional and multilayered concept that measures “an individual’s perception of his or her place in life” (38). Quality of life potentially consists of four basic dimensions, the two dimensions of health and economic status are objective, and the two dimensions of social relationships and emotions reflect individual judgments (39). Specific aspects of attitude toward aging (e.g., physical changes and psychological growth) are strongly associated with the quality of life of middle-aged and older adults (40). As Low argues, “the four dimensions of quality of life can influence mental health and thus change subjective perceptions of physical, cognitive and social aging” (41). Appropriate physical activity can help middle-aged and older women achieve good physical functioning, promote psychological well-being, and effectively improve attitude toward aging (42). A correlation between aerobic exercise and quality of life exists. For example, interventions such as square dancing, yoga and Tai Chi have shown small to moderate improvements in both physiological and psychological health for individuals aged 60 and above (43). Additionally, square dance helps enhance quality of life to some extent by providing social opportunities, which in turn boosts self-esteem and life satisfaction (44). However, yoga is more effective than square dance in improving menopausal symptoms, depression, and anxiety in middle-aged and older women (45).

The main characteristics of quality of life are health and well-being, which are key indicators of successful aging (46). Socioemotional choice theory reveals that as people age, they prioritize goals that are more emotionally meaningful and are motivated to fulfil their social preferences (47). In China, middle-aged and older women prioritize psychological values such as social relationships and emotions, while being motivated to persist in activities that are appropriate for them to meet their emotional needs (48). Square dancing creates a social environment that meets needs through interpersonal communication and significantly improves mood, promotes psychological adaptation, and enhances well-being (49).

In conclusion, square dance exercise improves middle-aged and older women’s perspectives on their overall mental health, including their attitude toward aging and their perceptions of quality of life.

### 1.4 The serial multiple mediation mechanism of loneliness and quality of life

The main theory of loneliness, the “Cognitive Discrepancy Model,” posits that loneliness arises when the ideal social relationships fail to match the actual social relationships in both quality and quantity (50). Greater participation in group-based leisure activities is associated with better health perceptions and closer social relationships among middle-aged and older adults (51). The sense of belonging generated by participation in group activities can be seen as a motivation to connect with society and others (52). It can also improve the collective well-being of middle-aged and older adults, allowing them to fully experience social relationships, reduce loneliness, and improve subjective well-being (53). Adequate social relationships can determine personal success related to happiness and quality of life (54). Reducing loneliness leads to a better self-perception of mental health, which in turn results in greater happiness, self-esteem and quality of life (55).

Loneliness correlates with the quality of life: the stronger the feelings of loneliness, the poorer the quality of life (56). Loneliness affects quality of life in two ways. Participation in group, recreational and social square dance exercises for people aged 45 and above has a more obvious effect on improving the loneliness of middle-aged and older adults, especially in the areas of social isolation and self-enclosure, which can further improve the overall quality of life (57). Square dancing is effective as a protective and accepting intervention after experiencing empty nesting and retirement, effectively linking social support to improved quality of life and positive attitude toward aging (21).

Active aging is a process of optimizing health, participation and security opportunities, aiming to improve quality of life as people age and create a favorable social environment that encourages middle-aged and older adults to engage in meaningful social activities (3). Additionally, the theory of aging activity suggests that when middle-aged and older adults remain active and maintain social interactions, they successfully transition into old age, reducing loneliness, delaying the aging process, and enhancing quality of life (58). Square dance has a strong predictive effect on increasing social interaction, improving physical function, establishing positive and optimistic psychological emotions, and fostering a positive attitude toward aging in middle-aged and older women. Therefore, we should actively pay attention to the attitude toward aging of middle-aged and older adults, and improving loneliness and quality of life may help reduce depression and anxiety and produce positive emotions (59).

In conclusion, loneliness and quality of life may serve as significant mediating variables between square dance exercise and attitude toward aging. However, this relationship needs further verification, especially in middle-aged and older female populations.

### 1.5 The present study

**Figure 1:**
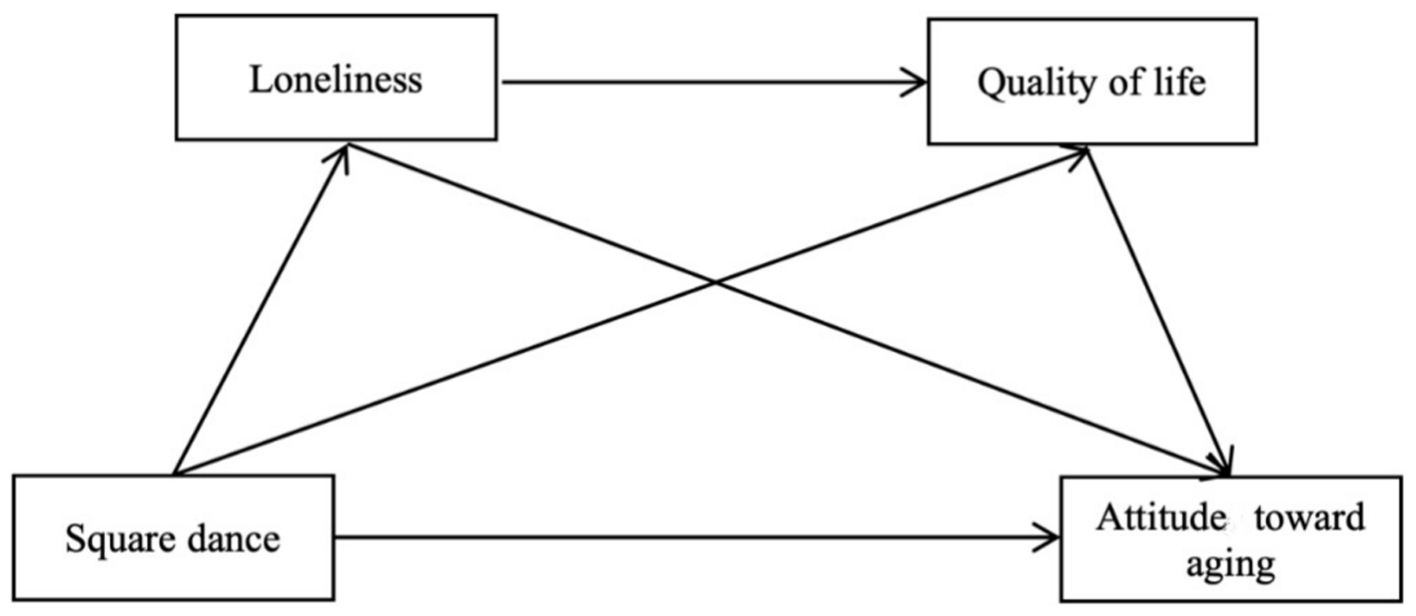
Research hypothesis model.

In recent years, physical and psychological problems have become a major global concern related to ensuring quality of life for a growing number of middle-aged and older women (60). Previous research related to square dance exercise has focused on its effects on the physical and mental health of older adults (61, 62). However, it is unclear whether square dance exercise improves attitude toward aging, and studies using loneliness and quality of life as mediating variables are relatively limited. Few cross-sectional surveys have been conducted on large samples of square dance participants. Therefore, the purpose of this study was to examine the relationship between square dance exercise and attitude toward aging among middle-aged and older adults, using loneliness and quality of life as the underlying mechanisms. The aim was to identify aspirations for active aging and to examine the relationship between attitudes in later life and loneliness and quality of life in the sport of square dancing. Based on the results, a foundation of information and recommendations will be provided for future square dance interventions and practices for middle-aged and older adults. The following research hypotheses were formulated (Figure 1):

*H1.* Square dance exercise predicts attitude toward aging in middle-aged and older women.

*H2.* Loneliness mediates the relationship between square dance exercise and attitude toward aging.

*H3.* Quality of life mediates the association between square dance exercise and attitude toward aging.

*H4.* Loneliness and quality of life chain-mediate the relationship between square dance and attitude toward aging.

### 1.6 Theoretical framework

This study employs 6 key theoretical perspectives to explore the relationship between square dance exercise and attitude toward aging, with a focus on the mediating roles of loneliness and quality of life. These theories collectively highlight the positive impact of square dancing on middle-aged and older women. Role theory suggests that square dance helps women redefine their social roles and identity during the aging process. Ecosystem theory emphasizes square dance as a cultural phenomenon that promotes personal well-being and enriches urban and community culture. Social–emotional choice theory posits that square dance provides emotional support, helping women focus on positive emotions and reduce stress. Social interaction theory highlights how square dance fosters social connections, improving psychological and social adaptability. The cognitive discrepancy model of loneliness suggests that square dance provides opportunities for women to form meaningful social relationships, reducing loneliness. Lastly, aging activity theory suggests that square dance, as a low-barrier activity, promotes physical health, social interaction, and psychological well-being, helping delay aging, improving quality of life, and fostering a positive attitude toward aging.

## 2 Materials and methods

### 2.1 Participants

The data were collected in 2023 through a web-based survey^1^ between August 9 and September 2, 2023, through the Questionnaire Star platform. We monitored the IP addresses of the respondents to avoid multiple repetitions. The questionnaires were distributed in 32 provinces, autonomous regions, and municipalities directly under the central government, including Beijing, Jiangsu, Guangdong, Fujian, Inner Mongolia, Shanghai, Qinghai, Shandong, Yunnan, and 32 other provinces, with a wide geographical distribution.

Since the data were self-reported by the participants, outliers may exist due to input errors or recall bias. To minimize the impact of outliers on data accuracy, SPSS 26.0 software was used to clean the data against the scale. The initial data set consisted of 5,068 respondents. After screening, 249 invalid questionnaires were excluded. Among these, 240 were from male respondents, including one who reported their age incorrectly. Additionally, 5 female respondents were excluded due to incorrect age reporting. Furthermore, 4 incomplete questionnaires were removed, along with those that showed repeated answers for given scales or displayed obvious response patterns. This process resulted in a final sample of 4,819 valid questionnaires, achieving a valid return rate of 95.08%. The age range of the sample for this study was 52.2% 45–59 years and 47.8% over 60 years.

### 2.2 Measures

- Demographic Information – The demographic information obtained by the questionnaire included the participants’ sex, age, residency status, place of residence, education level, whether they were enrolled in health insurance or pension insurance, and marital status, as detailed in Table 1.
- The Physical Activity Rating Scale-3 (PARS-3) assesses square dance movement to determine how extensively individuals are participating in the square dance movement. This scale was revised by Liang Deqing, who concluded that the scale has high reliability and validity (63). The scale has only three items, which are scored on a five-point scale ranging from 1 to 5, examining the amount of exercise in terms of intensity, time and frequency of participation in physical activity. The scoring method was as follows: physical activity = intensity × (time-1) frequency, with the highest score being 100 and the lowest score being 0. The scale evaluates exercise level as follows: low exercise level ≤ 19 points, moderate exercise level = 20–42 points, and high exercise level ≥ 43 points. In this study, the Cronbach coefficient was 0.645.
- Attitudes to Aging Scale – The Attitudes to Aging Questionnaire (AAQ) was developed by Laidlaw et al. in 2007 to measure older people’s evaluations and perceptions of themselves in the aging process using a self-report format (14). The questionnaire was introduced and adopted to the Chinese context by Huang Yifan in 2010 to create the Chinese version of the AAQ. The questionnaire includes three dimensions, i.e., psychosocial loss, coping with physical changes, and psychological gain, with 8 entries for each dimension and a total of 24 entries. The questionnaire was scored on a 5-point Likert scale ranging from 1 to 5, indicating “totally disagree” to “totally agree,” with the psychosocial loss dimension reverse scored. The total score of the questionnaire ranged from 24 to 120, with higher scores indicating more positive attitude toward aging. The Cronbach’s *α* coefficient of the questionnaire is 0.840, which indicates that the questionnaire has good reliability and validity and is suitable for the older population in China. In this study, the Chinese version of the AAQ measured the attitudes of older adults in the community, and the Cronbach’s α of the AAQ was 0.926.
- Emotional and Social Loneliness Scale – The Emotional and Social Loneliness Scale is based on the theory of R. S. Weiss and was developed by Wittenberg et al. in 1986. The scale consists of 10 items, with 5 items each for social and emotional loneliness (64). Each entry is scored on a 5-point scale ranging from 1 (none) to 5 (often), with higher scores indicating greater loneliness. This scale has been shown to have good reliability and validity. In this study, the Cronbach’s *α* coefficient for the total scale was 0.854.
- Quality of Life Scale SF-12 (12-items Short Form Health Survey) is a 12-item short-form health survey of the 36-item short-form health survey (SF-36) commonly used internationally (65). The SF-12, is a universal scale and has the advantages of simple responses and less operation time than the SF-36, and the correlation between the SF-12 and SF-36 has been confirmed in the literature (66). There are 12 items with in 8 dimensions: general health, physical functioning, physical functioning, somatic pain, energy, social functioning, emotional functioning, and mental health. Each item is scored according to the corresponding option, with questions 1, 8, 9, and 10 reverse scored. The SF-12 calculates two total scores, the total physical score and the total psychological score; higher scores indicate better quality of life.

**Table 1:** Demographic information of the participants (*N* = 4,819).

	Contents	*N* = 4,819	Percent (%)
Sex	Female	4,819	100
Age (in years)	45 ~ 59	2,516	52.2
	≥ 60	2,303	47.8
Living condition	Live with children	784	16.27
	Live with spouse and children	1,600	33.2
	Live with spouse	2,112	43.83
	Other	136	2.82
	Live alone	187	3.88
Place of residence	Countryside	1,008	20.92
	City	3,811	79.08
Education level and degree	Primary and below	308	6.39
	Junior high school	1749	36.29
	Senior high school includes technical secondary school	1822	37.81
	Bachelor degree or above (including Junior college)	940	19.51
Pension or medical insurance	Both	4,218	87.53
	Pension	353	7.32
	Medical	248	5.15
Marital status	Married	4,580	95.04
	Unmarried	24	0.5
	Divorce	215	4.46

Since the 12 questions of the quality of life scale have different value ranges, neither Cronbach’s *α* coefficients nor factor analysis are suitable for analysis, and the only way to test the reliability of the questionnaire is through retest reliability. However, due to the large number of people in this survey and considering the high cost of evaluating retest reliability, we did not evaluate retest reliability; however, a retest reliability analysis has been conducted for this scale, and the result was 0.864 (67). Therefore, it is reasonable to assume that the results of this scale are reliable and can be used for further analysis.

### 2.3 Statistical analysis

The predictor variable in this study was square dance exercise, the mediator variables were loneliness and quality of life, and the outcome variable was attitude toward aging. SPSS 26.0 and Process 4.0 were used to analyze the data statistically, with *α* = 0.05 indicating statistical significance. SPSS 26.0 statistical software was used to calculate and describe the distribution of the variables and their correlations; 95% confidence intervals for the mediating paths were tested using the bootstrap method, with 5,000 repetitions of the sample, and if the interval did not contain zero, the mediating paths were considered significant.

## 3 Results

### 3.1 Preliminary results

A total of 4,819 older women were evaluated in this study. Table 1 shows the demographic information, means and standard deviations of the participants’ sex, age, living condition, place of residents, highest level of education, pension or medical insurance, marital status. As shown in Table 1, the age of the 4,819 study participants was mainly over 45 years old. Among them, the number of females who ages 45 ~ 59 accounted for 52.2% of the total number, and who ages above 60 years old accounted for 47.8% of the total number. In terms of living condition, 784 people live with their children, accounting for 16.27% of the sample; 1,600 people live with spouse and children, accounting for 33.2%; 2,112 people live with spouse, accounting for 43.83%; 136 live with others, accounting for 2.82%; and 187 live alone, accounting for 3.88%. So, it can be seen that most women who participate in the activity prefer to live with others, like children, spouse and so on. Only a very small number of people choose to live alone.

As regards the place of residence, 3,811 participants live in the city, accounting for 79.08%; only 1,008 people live in the countryside, accounting for 20.92%. We can infer that most square dancing participants live in the city.

As to the education level and degree, 308 women only have primary education level even below, accounting for 6.39%; 1,749 have junior high school level, accounting for 36.29%; 1,822 women have senior high school includes technical secondary school level, accounting for 37.81%; 940 women have bachelor degree or above (including Junior college) level, accounting for 19.51%. So, it is clear that most participants only have below bachelor degree or above (including Junior college) education level.

In terms of the pension or medical insurance, 4,218 both have two, accounting for 87.53%; 353 only have pension, accounting for 7.32%; 248 only have medical, accounting for 5.15%. The number of having both is the most, and is much more than only have pension or medical.

As for marital status, the married group consists of 4,580 individuals, accounting for 95.04%, while the Unmarried group includes only 24 individuals, making up 0.5%. Additionally, the divorced group consists of 215 individuals, representing 4.46% of the total population.

### 3.2 Common method bias test

When self-reported methods are used to collect data, the issue of common method bias may arise. Common method bias was tested using the Harman one-factor test. The results showed that there was a total of one factor with an eigenroot greater than 1. The total variance explained by the first common factor was 39.867%, which is less than the critical value of 40%. Therefore, there was no common method bias in the data of this study.

Since the data were self-reported by the participants, outliers may exist due to input errors or recall bias. To minimize the biases typically associated with self-reported data, several methods were implemented. First, we used SPSS 26.0 software for data cleaning, identifying and reviewing outliers and inconsistent data to ensure the dataset’s reliability. Outliers and unlikely responses were excluded, enhancing the overall quality of the data. We also employed standardized, validated questionnaires to reduce response biases and ensure consistency across participants’ answers. This approach helped minimize interpretation errors and made the data more comparable and reliable. To address recall bias, we limited the recall period for certain questions, focusing on recent experiences and encouraging participants to report based on their current feelings or actions, thus reducing inaccuracies due to distant memories. Additionally, to mitigate social desirability bias, we assured participants of their anonymity and confidentiality, promoting honest and unbiased responses, particularly in sensitive areas. These combined strategies helped ensure the accuracy and integrity of the self-reported data.

### 3.3 Related analysis

The mean value, standard deviation and Pearson correlation analysis of each variable are shown in Table 2. Square dance exercise and quality of life (*r* = −0.267–0.127, *p* < 0.01) and attitude toward aging (*r* = 0.105–0.599, *p* < 0.01) were significantly negatively correlated with loneliness (*r* = −0.069–0.413, *p* < 0.01). The above results show that this approach is suitable for further analysis of intermediary effects.

**Table 2:** Correlations between variables (*N* = 4,819).

Variables	M	SD	1	2	3	4	5	6	7	8
Square dance exercise	25.888	17.87	1							
Physical health	37.023	5.294	0.127**	1						
Mental health	61.141	8.264	0.103**	−0.267**	1					
Psychosocial loss	28.318	8.952	0.096**	0.095**	0.238**	1				
Body change	32.244	6.711	0.169**	0.142**	0.241**	0.233**	1			
Psychological acquisition	25.713	8.572	0.105**	0.114**	0.112**	0.439**	0.599**	1		
Social loneliness	10.13	4.086	−0.069**	−0.056**	−0.301**	−0.189**	−0.076**	−0.039**	1	
Emotional loneliness	11.822	4.139	−0.013	−0.083**	−0.121**	−0.139**	−0.026	−0.006	0.413**	1

* *p* < 0.05, ** *p* < 0.01.

### 3.4 Testing the mediating effect

The mediation analysis was performed using the PROCESS plug-in in SPSS software, and the regression analysis in SPSS software was used to generate Model 1 (regression model of the independent variable on the dependent variable), Model 2 (regression model of the independent variable on the mediator variable 1), and Model 3 (regression model of the independent variable on the mediator variable 2) at the same time. The above hierarchical regression table is composed of Model 1, Model 2, Model 3, and PROCESS—generated Model 4 (multivariate regression model of the independent variable and the mediator variable on the dependent variable at the same time), and we can see the specific information of the individual models, as well as path coefficients a and path coefficients b in the mediator model, which are analyzed as follows.

In Model 1, a significant positive effect of square dance exercise on attitude toward aging (*β* = 0.162, *p* < 0.05), which means that c (total effect) in the mediation model is significant; that is, it is significantly different from 0 (not equal to 0).

In Model 2, square dance exercise had a significant negative effect on loneliness (*β* = −0.019, *p* < 0.05); in Model 3, square dance exercise had a significant positive effect on quality of life (*β* = 0.079, *p* < 0.05); and square dance exercise had a significant negative effect on loneliness (*β* = −0.353, *p* < 0.05).

Model 4 adds mediating variables based on Model 1. At this time, square dance exercise has a notable positive impact on attitude toward aging (*β* = 0.103, *p* < 0.05); that is, c’ (direct effect) in the mediating model is significant, as it is significantly different from 0 (not equal to 0); loneliness has a significant negative effect on attitude toward aging (*β* = −0.086, *p* < 0.05); and quality of life has a significant negative impact on attitude toward aging (*β* = −0.676, *p* < 0.05).

The 95% confidence interval for the mediating path was tested using the bootstrap method; if the interval did not contain 0, the mediating path was considered significant. The details of results of sequential mediation model of square dance and attitude toward aging were summarized in Table 3.

**Table 3:** Regression analysis of square dance, loneliness, quality of life and attitude toward aging in middle-aged and older women in China (*N* = 4,819).

	Attitude toward aging			Loneliness			Quality of life			Attitude toward aging		
	*β*	SE	*t*	*β*	SE	*t*	*β*	SE	*t*	*β*	SE	*t*
Constant	82.082**	0.478	171.88	22.434**	0.175	128.093	103.868**	0.428	242.533	19.189**	3.463	5.542
Square dance	0.162**	0.015	10.667	−0.019**	0.006	−3.346	0.079**	0.006	12.148	0.103**	0.015	6.998
Loneliness							−0.353**	0.017	−21.026	−0.086*	0.039	−2.204
Quality of life										0.676**	0.032	21.078
*R* ^2^	0.023			0.002			0.113			0.118		
*F*	113.79			11.199			307.847			215.275		

* *p* < 0.05, ** *p* < 0.01.

The bootstrap test further indicated that the direct effect was 63.46%, the mediating effect consisted of the indirect effect generated by the 3 paths (36.54% of the total effect), and the 95% confidence intervals of the three paths did not include 0, indicating that all three indirect effects reached a significant level. Path 1, square dance exercise → loneliness → attitude toward aging was composed of mediating effect 2 (0.0016); path 2, square dance exercise → quality of life → attitude toward aging was composed of mediating effect 3 (0.0533); and path 3, square dance exercise → loneliness → quality of life → attitude toward aging was composed of mediating effect 4 (0.0044). Accordingly, square dance exercise not only directly predicted attitude toward aging but also indirectly predicted attitude toward aging through the independent mediating effects of loneliness and quality of life, as well as through the chain mediating effect between loneliness and quality of life.

Finally, comparing the variability of the mediating effects of the 3 paths revealed that the mediating effect of path 1 was greater than those of path 2 and path 3. This suggests that quality of life plays a more important role in the relationship between square dance exercise and attitude toward aging. The details of results of effect values of the mediation effect tested by bootstrapping were summarized in Table 4.

**Table 4:** Analysis of mediation effects between square dance, loneliness, quality of life and attitude toward aging in middle-aged and older women in China (*N* = 4,819).

Intermediary process	Effect value	Bootstrpped LLCI^a^	Bootstrpped ULCI^a^	Effect size (%)
Square dance⇒Loneliness⇒Attitude toward aging	0.0016	0.0001	0.0041	0.99
Square dance⇒Quality of life⇒Attitude toward aging	0.0533	0.0429	0.0642	32.84
Square dance⇒Loneliness⇒Quality of life⇒Attitude toward aging	0.0044	0.0017	0.0073	2.71
Direct effect	0.103	0.074	0.131	63.46
Indirect effect	0.0593	0.044	0.067	36.54
Total effect	0.1623	0.132	0.192	100.00

a Boot LLCI and Boot ULCI refer to the 95% confidence intervals of the indirect effect estimated via the bias-corrected bootstrap method and refer to the upper and lower limits of the intervals, respectively. CI, confidence interval. Total effect = indirect effect + direct effect.

**Figure 2:**
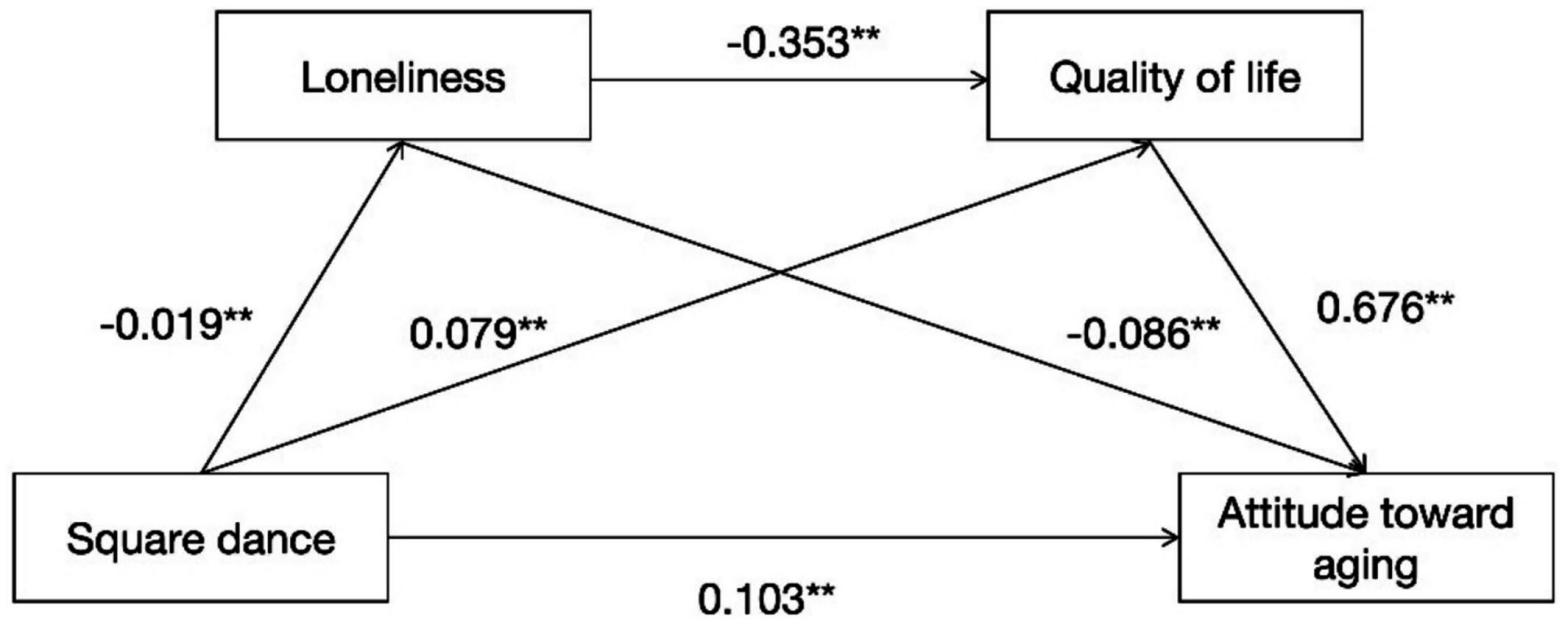
Chain mediating effect model of loneliness and quality of life between square dance exercise and attitude toward aging (*N* = 4,819). * *p* < 0.05, ** *p* < 0.01.

The path diagram in Figure 2 shows that (1) square dance exercise (*β* = 0.103**, *p* < 0.001) significantly and positively predicts attitude toward aging, indicating that the greater the level of square dance exercise is, the more positive the attitudes of older adults toward aging. (2) square dance exercise (*β* = −0.019**, *p* < 0.001) significantly and negatively predicts loneliness, indicating that the greater the level of square dance exercise is, the lower the loneliness felt by older people. (3) square dance exercise (*β* = 0.079**, *p* < 0.001) significantly and positively predicts quality of life, indicating that the greater the level of square dance exercise is, the greater the quality of life. (4) loneliness (*β* = −0.086**, *p* < 0.001) and quality of life (*β* = 0.676**, *p* < 0.001) significantly negatively and positively predict attitude toward aging, respectively. (5) loneliness (*β* = −0.353**, *p* < 0.001) significantly and negatively predicted quality of life, indicating that the lower the loneliness was, the greater the quality of life of the older participants.

The corrected bias bootstrap method with 5,000 replicated samples was used to test each path of the serial mediation model, and the mediation effect of loneliness was 0.0016, 95% CI [0.0001, 0.0041], with an effect size of 0.99%, and the mediation effect of quality of life was 0.0533, 95% confidence interval [0.0429, 0.0642], with an effect size of 32.84%; its series mediated effect was 0.0044, 95% confidence interval [0.0017, 0.0073], and its effect size was 2.71%.

In conclusion, loneliness and quality of life play a serial-mediated role in the relationship between square dance exercise and attitude toward aging. The details of each path case of the model are shown in Table 4.

## 4 Discussion

In a cross-sectional survey of 4,819 middle-aged and older women in China, this study aimed to explore the relationship between square dance exercise and attitude toward aging and to reveal the mediating roles of loneliness and quality of life in the relationship between square dance exercise and attitude toward aging. Interventions that increase opportunities for social interaction versus improving social skills are more effective in reducing overall loneliness (68). At the same time, quality of life is closely related to physical, psychological and social adaptation. Therefore, square dance exercise is characterized by a wide range of groups, rich socialization, and diverse movements that comprehensively regulate the physical and mental functions of middle-aged and older women, thus improving their attitude toward aging. This study supports the significant positive predictive effect of square dance exercise on attitude toward aging, as well as the formation of a chained mediation model, validating H1, H2, H3, and H4.

### 4.1 The direct relationship between square dance and attitude toward aging

Physical activity (PA) is a major component of maintaining health and well-being (69). PA is a positive predictor of attitude toward aging, which is consistent with previous research findings (70). PA is considered to be a part of active lifestyle and necessary condition for active aging. It promotes healthy aging by facilitating the maintenance and improvement of personal fitness (71), enhancing the general self-efficacy of middle-aged and older adults, and improving the quality of life of middle-aged and older adults so that they can live healthier, more fulfilling lives (72).

Square dance exercise significantly positively predicts attitude toward aging, which confirms the hypothesis of this study. The social choice emotion theory further explains the perception of survival time in middle-aged and old adults, who are more inclined to emotion management, obtain intimate emotional experience, and pursue positive emotions (18). With the gradual increase of age, the middle-aged and older population will have a negative attitude toward aging along with the loss of physical, psychological, and social fields (73). In the city, because in the context of sports power and national fitness, residents have more access to cutting-edge policies, so they are more affected by policies and have higher enthusiasm to participate in sports activities. In addition, cities have more funds than rural areas, so they are more likely to have access to high-quality sports resources and have a stronger incentive to participate in sports activities. In summary, it can be seen that urban residents are more aware of individual health. Middle-aged and older adults’ awareness of the importance of recreational and cultural activities, and the support of family and friends enhancing their eagerness, attitude, and motivation to participate in PA. What underpins their practice is the increased awareness of their own health and their awareness of the value of “aging well” (74).

Square dance exercises can enable middle-aged and older adults to establish stable and intimate relationships with team members in the process of long-term participation, so as to produce a more positive emotional experience in the team. This increased social interaction, coupled with positive personal emotional experiences, is a key factor in shaping positive attitude toward aging and promoting mechanisms for successful aging (19). Because of its low cost and easy participation, square dancing attracts middle-aged and older adults with low socioeconomic status. In this study, most of the participants in square dancing have only a secondary education level. This reflects the generally lower educational attainment of the generation born around the 1950s, and the less educated middle-aged and older adults may be more inclined to choose square dancing as a low-barrier way to socialize and exercise (75). Meanwhile, the percentage of those who participated in square dance exercise with a time intensity of 30 min or more was close to 90%. By increasing the duration of square dance exercises, the cardiopulmonary function can be enhanced, muscle strength and endurance can be improved, and the overall level of physical health can be improved (76). Furthermore, the musical rhythm of square dancing has a unique charm. As the rhythm of the music changes, middle-aged and older adults are immersed in the music melody, which can effectively promote the activity of the cerebral cortex and improve their cognitive function (77). At the same time, rhythmic exercise can trigger positive emotional experiences, such as happiness and pleasure, which is conducive to middle-aged and older adults to show greater energy and vitality, and achieve a positive aging attitude, and promote successful aging (78).

In summary, a strong correlation between square dance exercise and attitude toward aging. Attitude toward aging usually become more positive and result in a stable emotional experience, that promotes a positive attitude toward life in the middle-aged and older adults.

### 4.2 The intermediary role of loneliness

Loneliness plays an important role in the health and well-being of middle-aged and older adults (74). Square dance exercise was negatively correlated with both types of loneliness, and square dance exercise can contribute to attitude toward aging through both dimensions of loneliness (social loneliness and emotional loneliness). This confirms hypothesis 2 of this study. A lack of close relationships with spouses, children, and even grandchildren are associated with emotional loneliness. Thus, social loneliness is more general in nature, whereas emotional loneliness is more specific and varies with specific intimate connections with significant others. Physical activity was significantly negatively correlated with loneliness, with higher physical activity participation being associated with lower loneliness, which is consistent with previous research findings (79).

According to the effect analysis table of this study, the mediating effect size of loneliness in the relationship between square dance exercise and attitude toward aging was 0.99%. Square dance exercise enlarges the social circle of participants, satisfies the social needs of middle-aged and older women, and helps them overcome loneliness (80). However, the mediating effect of loneliness was weaker. First, 96.12% of this study’s sample living with their spouses and children. Adult children can provide support to their aging parents during their aging and retirement transitions, thereby reducing their parents’ fear and emotional distress about their future lives. At the same time, China is a society with a traditional culture of respecting the older and a country where the two-way support relationship between parents and children is very close (81). Family intergenerational support provided by children to the older, such as financial and emotional support, is regarded as an act of respecting the older and aligns with traditional Chinese filial piety norms (82). China’s close intergenerational support system, rooted in its tradition of respecting the older, allows adult children to alleviate their parents’ aging-related anxieties through financial and emotional support. This reflects a strict adherence to the norms of filial piety. Moreover, the degree of depression among the older adults ovaries significantly depending on their marital status. Married older adults have the lowest level of depression, followed by those who are divorced or widowed, while unmarried older adults have the highest level of depression (83). On the one hand, spouses can provide emotional support and comfort, whereas unmarried or widowed individuals lack such support and are more prone to feelings of loneliness and loss. On the other hand, couples can support each other and share burdens and stress, thereby reducing depression (84).

First, middle-aged and older women emphasize family support, and support from family members (e.g., spouses and adult children) plays an important role in maintaining their mental health (85). Women who are square dance exercisers are more likely to have relationships with their families and are less likely to feel lonely. Second, according to the data, the middle-aged and older adults who participate in square dance exercise are mostly urban residents, accounting for 79.1% of the total population. The middle-aged and older adults living in urban areas have rich daily lives, better public facilities and richer urbanization resources. The healthful role played by urban and community design that promotes physical activity may moderate declines in physical function. Loneliness is lower in urban-dwelling middle-aged and older adults. Third, social interaction theory suggests that social ties and interpersonal contact are the main reasons for participation in group movements. Physical activity can facilitate social relationships, thereby improving an individual’s mental health. Square dance is a form of daily group exercise, providing middle-aged and older women with a relaxing and enjoyable exercise atmosphere and the opportunity to expand their social interactions during group exercise (15). Square dance can effectively compensate for the social isolation and negative emotional problems caused by socialization deficits (86).

In this study, emotional loneliness is not significantly associated with square dance exercise, changes in attitude toward aging, or loss of psychological identity. Because this study focused on a group of middle-aged and older women, there were fewer effects on emotional loneliness, which led to a weaker mediating effect on loneliness. Loneliness has a low impact as a mediator.

### 4.3 The intermediary role of quality of life

Quality of life is a conscious cognitive judgment of life satisfaction that accounts for the physical, psychological and social aspects of life (87). Square dance exercise is positively correlated with two dimensions of quality of life (physical health, mental health) and three dimensions of attitude toward aging (psychosocial loss, physical change, and psychological gain). This confirms H3 of this study. Higher levels of physical activity are independently associated with long-term quality of life, according to previous research (88). Regular physical exercise not only improves physical condition but also adjusts the process of self-adaptation to the social environment and helps middle-aged and older women competently regulate their psychology and cultivate good psychological quality by strengthening their communication with the outside world.

Inequalities in health are exacerbated by unequal socioeconomic status, which reflects a range of key social determinants of people’s health and is a root cause of health disparities (89). China is a vast country with large differences in economic development across the country, and differences exist in the factors affecting quality of life in different places and at different stages of development (90). The impact of economic development on mind and body shows different patterns in urban and rural areas. The impact of economic conditions on the quality of life of the older adults in urban areas is relatively weak due to the higher level of urban economy, superior urban planning, better medical conditions and supporting facilities. From the perspective of demand theory, non-material needs become important when economic resources mainly satisfy the material needs of the population (91).

According to the decomposition of the mediating effects in this paper, 32.84% of the effects were mediated through quality of life, which was more significant than the effect of loneliness. In the domain of physical health, square dance exercise can significantly improve the physical health of middle-aged and older women. Long-term adherence to participate in square dance exercise can affect the body’s joints, increase core strength, coordination of the upper and lower limbs, increase cardiorespiratory endurance, effectively reduce muscle and bone loss, improve body shape and strengthen the immune system (61). However, as square dancing is a spontaneous sport, the lack of professional risk awareness and supervision of participants may lead to varying degrees of injuries among middle-aged and older adults, affecting their quality of life (92).

First, from the perspective of mental health, square dance exercise is accompanied by music, which allows middle-aged and older women to promote the secretion of dopamine neurotransmitters. A pleasant and relaxing atmosphere enables them to experience the joyful experience of “reliving,” reduce anxiety and tension in life (93). As a result, they actively choose to spend their time on square dance workouts. Second, square dance exercise can promote communication and exchange between middle-aged and older women, promote the development of interpersonal relationships and help middle-aged and older women actively integrate into society and regain their own self-worth. Social–emotional selection theory also suggests that the socially meaningful attributes of this activity appeal to middle-aged and older women (18). However, it is verifiable that Tai Chi, yoga, and other forms of exercise are significantly better than square dancing for cognitive function and mood in middle-aged and older adults (22).

Attitude toward aging are an important predictor of quality of life and one of the most important factors that enables middle-aged and older women to age positively (15). The quality of life of middle-aged and older women can be improved through long-term adherence to square dance exercise, thereby increasing the likelihood of successful individual aging. As the cycle begins, behavior → quality of life → attitude toward aging form a closed loop (86). Participation in square dance exercise can be transformed into a positive experience of the physical, psychological and social environment. Improving middle-aged and older women’s perceptions and experiences of their aging and helping them face aging with a more positive and optimistic attitude is important for promoting successful aging.

### 4.4 The serial multiple mediation mechanism of loneliness and quality of life

This study also examined whether the relationship between square dance exercise and attitude toward aging was influenced by levels of loneliness and quality of life. However, the mediating roles of loneliness and quality of life in the relationship between square dance exercise and attitude toward aging have been rarely discussed in related studies. In this study, a negative correlation between loneliness and quality of life, indicating that the lower the sense of loneliness among middle-aged and older adults, the higher their quality of life. Quality of life, a good indicator of overall health, is more significantly affected by loneliness among older adults, which is consistent with H4 of this study. Participation in physical activity helps middle-aged and older women become more socially engaged and better integrated into society, which in turn produces psychological benefits, improves the quality of life of middle-aged and older women and reduces loneliness (94).

According to social identity theory, an individual’s sense of belonging to a group influences their perceptions of aging (95). Square dance provides middle-aged and older adults with opportunities to engage in group activities and experience social connections, fostering positive emotional experiences within the group. This increased social interaction and a strong sense of belonging are closely linked. Overall, square dance exercise positively impacts the quality of life of middle-aged and older adults by reducing feelings of loneliness, enhancing social closeness and promoting positive emotional experiences. This further proves the potential value of square dancing in promoting people’s positive attitude toward aging.

According to the mediating effect analysis in this paper, the mediating effect size of square dance exercise → loneliness → quality of life → attitude toward aging is 2.71%, which is not high. This may be because 79% of the middle-aged and older women in the sample in this study lived in the city. In 2011 China Longitudinal Healthy Longevity Survey (CLHLS), the mental health of urban older people is better than that of rural older people. This is essentially related to the fact that healthcare, social welfare, cultural and recreational facilities are better in urban areas. The standard of living and social status of the urban older adults is relatively high (96). Adequate health-care services, modern facilities, a sense of personal security and opportunities for active participation in social groups or organizations are the main reasons for the high quality of life of older adults in urban areas (97). In addition, the effect size of loneliness in this study was 0.99%. This may be related to the fact that the survey was conducted during the COVID-19 pandemic, a period when the virus inevitably impacted individuals’ psychological and emotional well-being, possibly leading to a decrease in social engagement (98). However, the social environment of the time, while hindering face-to-face social interactions, increased time spent with family. Over time, square dance workouts have been adapted to online home workout formats.

In conclusion, square dance as a group physical activity, not only helps reduce loneliness and improve the quality of life for middle-aged and older adults but also generates positive emotions that promotes a positive attitude toward aging. Additionally, loneliness and quality of life in middle-aged and older adults are influenced by various factors, including economic and cultural aspects (99). The sample in this study had relatively low levels of loneliness. This may explain why square dancing is not as effective in improving loneliness, which further affects the impact of square dancing on quality of life and attitude toward aging. Therefore, future research could conduct comparative analyses in areas with different levels of urbanization to explore the varying effects of square dance in different social environments.

### 4.5 Limitations and future research directions

This study also has some limitations. First, this study utilized a cross-sectional design, so interpretation of results should be considered with caution. To deepen the understanding of causality, researchers could use a longitudinal design to track changes between square dance exercise, loneliness, quality of life, and attitude toward aging. Second, the sample of this study was mainly focused on the middle-aged and older female population, which may have affected the overall understanding of the exercise effects of square dancing. Whether the results of future research can be generalized to other genders or age groups needs to be confirmed by further interventions and surveys to more accurately assess the impact of square dancing on the physical and mental effects of different populations. In addition, the samples included in this study are from China, and the effects of square dancing have not been studied abroad. Therefore, the results of this study can only be generalized to China, and for the time being, they are not applicable to foreign countries with different economic conditions and cultural backgrounds. Finally, in addition to the indicators in this study, other sociodemographic variables, such as level of education, presence of children or grandchildren, race and ethnicity, history of chronic disease, and other important psychological variables, such as anxiety, depression, motivation to exercise, and personality traits should be included in future related studies. The selection of variables and the construction of models, especially the influence of complex multidimensional concepts and potential factors on the relationships between variables, remain to be tested experimentally.

### 4.6 Implications

Theoretical implications: this study analyses how square dance exercise affects middle-aged and older women’s attitude toward aging with the help of social–emotional choice theory, social interaction theory, and aging activity theory and explores the chain-mediated model of loneliness and quality of life to further elucidate the intrinsic and extrinsic mechanisms involved. By giving middle-aged and older women specific social roles, it is possible, in a certain sense, to alleviate anxiety or stress caused by various factors such as retirement and aging, and to grow in physical and mental health. In summary, this study examined the effects of square dance exercise on middle-aged and older women’s attitude toward aging, providing a rich theoretical reference for empirical research on square dance exercise interventions for middle-aged and older women.

Practical implications: against the multifaceted and complex background of population aging, some middle-aged and older women experienced a certain degree of decline in their physical and mental health during the coronavirus epidemic. Spontaneous participation in group-type social activities, such as square dance exercise, can raise older people’s awareness of fitness and health, help them find a sense of belonging to a group, enhance their self-worth, and change their attitude toward aging. For this reason, the present intervention study on reducing loneliness and improving the quality of life of the middle-aged and older female population is of profound practical significance and is in line with the current background and trend of coping with population aging, providing a new intervention idea for achieving the goal of active aging.

## 5 Conclusion

Based on a cross-sectional design and utilizing a large sample empirical research methodology. This study provides an in-depth examination of the relationship between square dance exercise and attitude toward aging, with a particular focus on the intrinsic mediating role of the variables of loneliness and quality of life. First, square dance exercise is a positive predictor of attitude toward aging in middle-aged and older adults. Second, loneliness and quality of life significantly moderate the relationship between square dance exercise and attitude toward aging and mediate the effect through all three pathways together. Moreover, further analysis revealed that the mediating effect of quality of life is more significant than that of loneliness. In conclusion, this study aimed to reveal the reduction in loneliness and improvement in quality of life as potential mechanisms underlying the relationship between square dance exercise and attitude toward aging and to emphasize that positive attitude toward aging can be effectively promoted among middle-aged and older adults through square dance exercise. The positive effects of square dance exercise are worthy of further in-depth research and application promotion to make an important contribution to improving the quality of life and happiness of middle-aged and older adults and realizing the strategy of positive aging.

## Data Availability

The raw data supporting the conclusions of this article will be made available by the authors, without undue reservation.

## Footnotes

^1^ https://www.wjx.cn/
